# Phenotypic and genetic characterization of Africanized *Apis mellifera* colonies with natural tolerance to *Varroa destructor* and contrasting defensive behavior

**DOI:** 10.3389/finsc.2023.1175760

**Published:** 2023-08-31

**Authors:** Eliana Mariel Bianchi, Carolina Ferrari, Natalia C. Aguirre, Carla V. Filippi, Pablo A. Vera, Andrea Fabiana Puebla, Gerardo P. Gennari, Graciela A. Rodríguez, Alejandra Carla Scannapieco, Cintia V. Acuña, Silvia B. Lanzavecchia

**Affiliations:** ^1^ Área Animal, Instituto de Investigación Animal del Chaco-Semiárido (IIACS) - Instituto Nacional de Tecnología Agropecuaria (INTA), Santa Rosa de Leales, Tucumán, Argentina; ^2^ Escuela de Ciencias Agrarias, Naturales y Ambientales (ECANA), Universidad Nacional del Noroeste de Buenos Aires (UNNOBA), Pergamino, Buenos Aires, Argentina; ^3^ Instituto de Agrobiotecnología y Biología Molecular (IABIMO), Instituto Nacional de Tecnología Agropecuaria (INTA) - Consejo Nacional de Investigaciones Científicas y Técnicas (CONICET), Hurlingham, Buenos Aires, Argentina; ^4^ Laboratorio de Bioquímica, Departamento de Biología Vegetal, Facultad de Agronomía, Universidad de la República, Montevideo, Uruguay; ^5^ Unidad de Genómica, Instituto de Biotecnología-Instituto de Agrobiotecnología y Biología Molecular (IABIMO), Centro de Investigación en Ciencias Veterinarias y Agronómicas (CICVyA), Instituto Nacional de Tecnología Agropecuaria (INTA)-Consejo Nacional de Investigaciones Científicas y Técnicas (CONICET), Hurlingham, Buenos Aires, Argentina; ^6^ Estación Experimental Agropecuaria (EEA) Famaillá, Instituto Nacional de Tecnología Agropecuaria (INTA), Famaillá, Tucumán, Argentina; ^7^ Estación Experimental Agropecuaria (EEA) Ascasubi, Instituto Nacional de Tecnología Agropecuaria (INTA), Hilario Ascasubi, Buenos Aires, Argentina; ^8^ Instituto de Genética, Instituto Nacional de Tecnología Agropecuaria (INTA), Instituto de Agrobiotecnología y Biología Molecular (IABIMO) Consejo Nacional de Investigaciones Científicas y Técnicas (CONICET), Hurlingham, Buenos Aires, Argentina

**Keywords:** Africanized honey bee, defensiveness, next generation sequencing techniques, ddRADseq, breeding program

## Abstract

Africanized *Apis mellifera* colonies with promising characteristics for beekeeping have been detected in northern Argentina (subtropical climate) and are considered of interest for breeding programs. Integral evaluation of this feral material revealed high colony strength and resistance/tolerance to brood diseases. However, these Africanized honeybees (AHB) also showed variable negative behavioral traits for beekeeping, such as defensiveness, tendency to swarm and avoidance behavior. We developed a protocol for the selection of AHB stocks based on defensive behavior and characterized contrasting colonies for this trait using NGS technologies. For this purpose, population and behavioral parameters were surveyed throughout a beekeeping season in nine daughter colonies obtained from a mother colony (A1 mitochondrial haplotype) with valuable characteristics (tolerance to the mite *Varroa destructor*, high colony strength and low defensiveness). A Defensive Behavior Index was developed and tested in the colonies under study. Mother and two daughter colonies displaying contrasting defensive behavior were analyzed by ddRADseq. High-quality DNA samples were obtained from 16 workers of each colony. Six pooled samples, including two replicates of each of the three colonies, were processed. A total of 12,971 SNPs were detected against the reference genome of *A. mellifera*, 142 of which showed significant differences between colonies. We detected SNPs in coding regions, lncRNA, miRNA, rRNA, tRNA, among others. From the original data set, we also identified 647 SNPs located in protein-coding regions, 128 of which are related to 21 genes previously associated with defensive behavior, such as *dop3* and *dopR2*, *CaMKII* and *ADAR*, *obp9* and *obp10*, and members of the 5-HT family. We discuss the obtained results by considering the influence of polyandry and paternal lineages on the defensive behavior in AHB and provide baseline information to use this innovative molecular approach, ddRADseq, to assist in the selection and evaluation of honey bee stocks showing low defensive behavior for commercial uses.

## 1 Introduction

During the16th century, the European honey bee, *Apis mellifera* L. (Hymenoptera: Apidae), was introduced into America for beekeeping purposes (1–3). The subspecies brought to the continent by European settlers were *Apis mellifera. mellifera* L, *A. m. ligustica* Spinola, *A. m. carnica* Pollmann*, A. m. caucasica* Pollmann*, A. m. lamarckii* Cockerell, *A. m. syriaca* Skorikov, *A. m. cypria* Pollmann, and *A. m. intermissa* Buttel-Reepen (2, 3). These subspecies have continued to be imported ever since, through the commercialization of fertilized queens and queen cells (4, 5). In turn, the first recorded entry of honey bees of African origin, *A. m. scutellata* Lepeletier, took place in 1956 (6) in the context of a genetic breeding program. Despite the controlled conditions of the program, an accidental escape of these African bees, favored by their colonization capacity and genetic dominance, dispersed in an uncontrolled manner throughout South America by crossing with honey bee populations of European origin, initiating a hybridization process known as “Africanization’’ (7–10). In Argentina, populations derived from African subspecies (*A. m. scutellata* and *A. m. intermissa*) and from the Iberian Peninsula (*A. m. iberiensis* Engel) have been registered and are mainly distributed in the northern region of the country. Particularly, the presence of the last two subspecies of *A. mellifera* suggests a second source of A and M lineages, respectively (11–13).

The Africanization process marked a milestone in American beekeeping since Africanized honey bee (AHB) colonies retained many advantageous productive traits of their African ancestors, such as active resistance to brood diseases and natural tolerance to pathogens and parasites (14–16), as well as high genetic variability, which favored the emergence of climate-adapted ecotypes (5, 9, 17, 18). However, they also exhibited disadvantageous characteristics such as high defensiveness and swarming tendency (19–23). In addition, AHB exhibited a lower productivity (weight of honey produced) compared with EHB (24, 25).

Given the threat of global warming and the growing need for food equity, AHB colonies are regarded as promising genetic resources for beekeeping, due to their shorter breeding cycle, compared to European honey bees (26, 27), and natural adaptation to subtropical and tropical climates (10, 28, 29). Nonetheless, as approximately 3% of the human population is allergic to honey bee venom (30, 31), the defensive behavior displayed by workers is a topic of interest not only for beekeeping but also for public health (32). Defensive behavior in honey bee colonies is defined as the individual and collective reactions of worker bees in response to external disturbances to the hive (33). It involves different actions such as persuasion (rapid flight, high-pitched buzzing, chasing, hitting, and biting), alarm pheromone release (34–36), and stinging as a direct attack action on the threat (37). This behavior has been described as a highly heritable trait, with genetic dominance and paternal effect (29, 38, 39). The level of defense response of a honey bee colony is related to its sensitivity to the alarm pheromone, visual stimuli, and propensity to sting, run or fly (40), and it requires a sophisticated mechanism of sensory integration, involving olfactory, visual and mechanosensory signals (33, 38). Due to the interest in defensive behavior, genetic studies have been carried out using molecular markers (40, 41), and expression analyses of candidate genes have allowed the identification of molecular pathways associated with the expression of this behavior (42, 43). Age, genetics and adaptation to the environment have been proposed as the main factors associated with defensive behavior in honey bees (44–47). In addition, Gibson et al. (48) have evidenced the existence of asymmetric allelic expression patterns in hybrid honey bees associated with maternal biases and epigenetic regulation (38, 49, 50), which would act as regulators of aggression expression.

Beekeeping is an important economic activity in Argentina, as this country is the fourth world producer and the third exporter of high-quality honeys (51). This commercial activity is mostly performed by small and medium-sized Argentine producers (52, 53). In support of beekeepers, Argentina has implemented honey bee breeding programs to select and multiply bee stocks of European origin with desirable characteristics of production and behavior (54). Particularly, the MeGA program (PROAPI-INTA) focuses on the selection and conservation of local stocks (mostly established in a temperate climate) with demonstrated tolerance or active resistance to mites and brood diseases, low defensiveness, and high productivity (54–56). Conversely, in northern regions of the country the presence of Africanized feral populations with undesirable characteristics for beekeeping (swarming tendency and high defensive behavior) precludes the breeding selection contributing to the genetic background of commercial honey bee colonies (5, 57). Recently, colonies with desirable traits (docility, resistance to brood disease, and adaptation to a subtropical climate) have been characterized in the northwestern region of the country (58, 59), offering an opportunity to preserve honey bee stocks for the development of sustainable apiculture in a subtropical climate. These hybrid colonies have possibly inherited the best characteristics from their European and African origins, as have previously been described in other regions of the American Continent (3, 60, 61).

Next Generation Sequencing (NGS) techniques enable the detection of large numbers of single nucleotide polymorphisms (SNPs) in a cost- and time-efficient manner (62). In *A. mellifera*, these technologies have facilitated high-resolution genomic studies at the population level, genetic variability and evolution analyses and genotype-phenotype relationship supporting marker-assisted selection for breeding (63–66). Within this group, the Double Digest Restriction-Site Associated DNA (ddRADseq) technique has been widely utilized as an affordable alternative for the genotyping of individuals in model or non-model species with a reduced representation of the genome of interest, thus lowering costs and analysis time (67). Several investigations have been carried out in the last decade using RADseq techniques in *A. mellifera* (63, 68), including evolutionary history studies (69) and analyses of population structure and variability (70–73).

In this article, we describe the integral characterization of AHB colonies of the same wild origin, located in northwest Argentina (Tucumán province). These colonies possess demonstrated tolerance to the ectoparasitic mite *Varroa destructor* Anderson and Trueman (Acari: Varroidae) and variability in defensive behavior (58). Evaluations were performed throughout the 2019–2021 productive beekeeping seasons and honey bee colonies with contrasting defensive behavior were analyzed by ddRADseq. The SNP analysis presented here provides a genetic characterization of these contrasting colonies and a set of markers and genomic regions potentially associated with defensive behavior to be further analyzed in breeding programs that seek to improve beekeeping using innovative NGS tools.

## 2 Materials and methods

### 2.1 Biological material

We selected a previously characterized *A. mellifera* colony (named LE2) (58), hereafter named LE0, as a mother colony to generate the biological material analyzed in the present study. The LE0 colony, established in the Leales Apiary (Santa Rosa de Leales, Tucumán, Argentina), represents a hybrid honey bee stock and possesses an Africanized mitochondrial lineage (A1). It has shown high colony strength in spring (category 1), as well as low defensiveness and the ability to survive medium-high levels of *V. destructor* without acaricide treatment since 2017 (58, 59).

**Figure 1:**
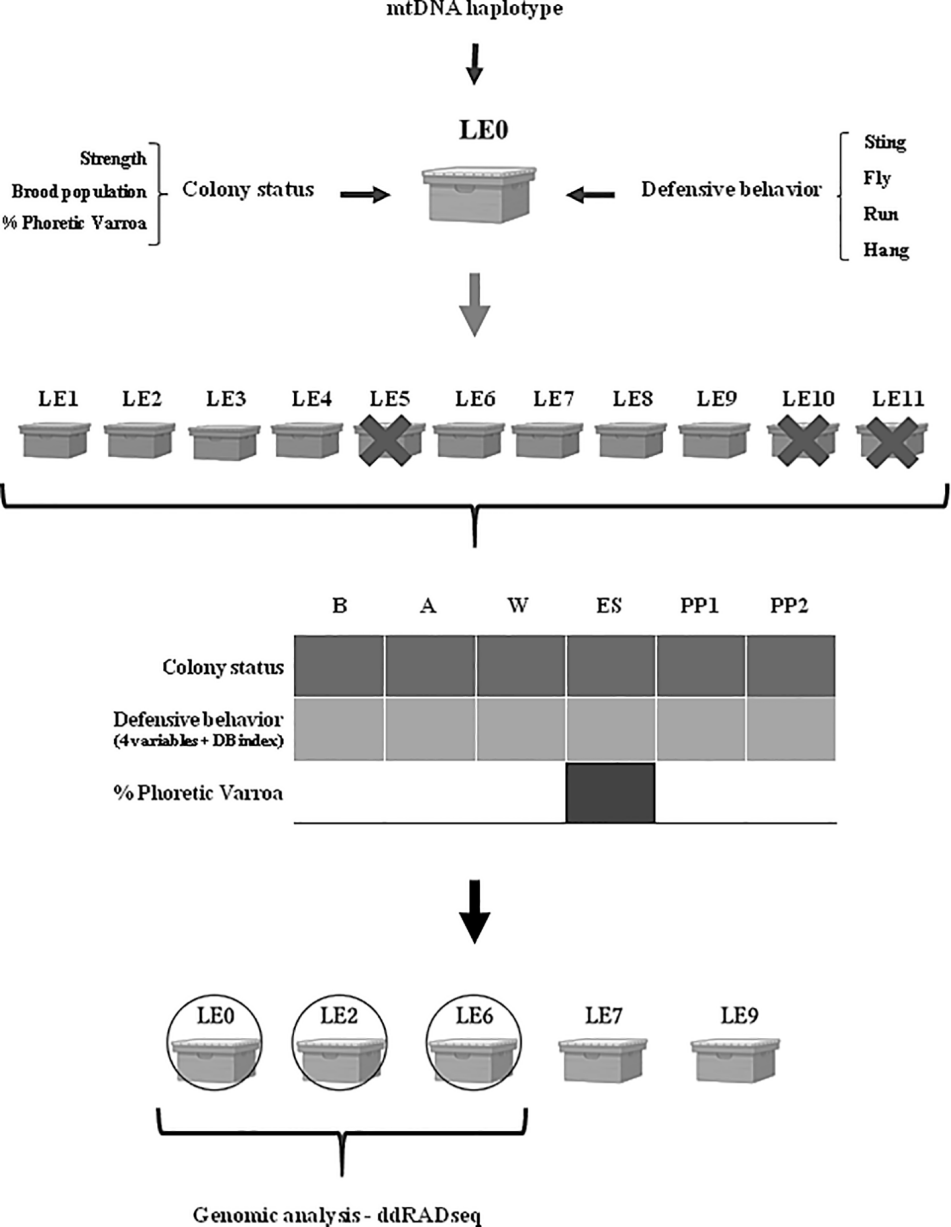
Diagram showing the selection process of hybrid A*. mellifera* colonies for obtaining the biological material for this study. The black arrow indicates selection of colonies; The grey arrow indicates multiplication. Mt. Haplotype: mitochondrial haplotype (genetic characterization). The colonies were surveyed six times during the beekeeping season: late spring-summer 2019, autumn 2020, winter 2020, early spring 2020, 1st productive peak 2020 and 2nd productive peak 2020. Crossed out colonies indicate lost colonies during the time of the survey.

Daughter queens were generated in September 2019 using the traslarvae method (74–76) and nucleus creation according to protocols established for the region (77, 78; **Figure 1**). Once natural fertilization of the new queens was confirmed, surviving daughter colonies (LE1-9), showing similar high strength status, and the mother colony LE0 were transferred to a new location to facilitate their management. This new apiary was established in Tafí Viejo (26°44’08.8’’S; 65°17’14.3’’W), Tucumán province (Argentina). In this region, the landscape corresponds to the piedmont type (79) with predominant commercial citrus production, followed by sugarcane cultivation (80).

### 2.2 Colony status measurements

The daughter colonies were established and after three months to strengthen (minimum adult population = at least three frames covered by bees) the survey started. Colony strength (adult population) was estimated by visual inspection of the top of the hive following specific protocols set up for honey bee stocks adapted to a subtropical climate (northern Argentina) (77, 81). The presence of hybrid honey bee colonies (mixed European and African lineages) in northern Argentina has been previously detected and characterized (5, 12, 57) and constitute baseline information for the evaluation proposed in the present study. Briefly, three categories of colony strength were assigned according to the number of hive frames covered with adult honey bees when the top of the beehive is opened: the category 1 is registered when at least seven frames of the hive are covered by bees; category 2 (five to seven frames); and category 3 (four or fewer frames).

The brood population was evaluated for each colony as the total area of combs covered with brood and the number of brood frames (82, 83). For the observations, the bee hives were opened, and the brood frames were sequentially removed. A panel subdivided into quadrants of equal size was superimposed on each brood frame to estimate the average area covered with brood. The number of brood frames fully covered with brood was also registered.

The level of phoretic Varroa was estimated using the “Ethanol wash method” (84, 85) only once during the beekeeping season: (ES) on winter surviving colonies (**Figure 1**). This method is based on the collection of 250 to 300 worker bees from brood frames in a jar containing 70% v/v ethanol. The bottle is then capped and shaken vigorously to dislodge mites from the bees, and then separated by filtering. The percentage of mites is calculated from the number of bees contained in the sample (% phoretic Varroa = mites/bees*100) (84, 85). This parameter was registered in the analyzed colonies with the purpose of confirming their tolerance to the ectoparasitic mite, *V. destructor*. All the colonies analyzed in the present study were maintained without acaricide treatment.

### 2.3 Defensive behavior

Defensive behavior was assessed by opening each honey bee hive with minimal application of smoke and subsequent direct observation for 30 seconds according to Ávalos et al. (86). Four defensive behavior variables were registered: “run” (tendency of worker bees to run on honey bee combs), “fly” (tendency of worker bees to fly during honey bee hive colony manipulation), “sting” (tendency of worker bees to hit the operator) and “hang” (tendency of worker bees to be grouped over the honey bee combs). A score range from 1 to 4 (1 = the lowest intensity of response; 4 = the highest intensity of response) was assigned to each of the four behavior variables measured. All the observations were performed and registered by the same operator. In addition, at each time point, to eliminate the effect of the presence of the alarm pheromones released by guarding honey bee workers (36, 87, 88), the behavior of half of the colonies was measured intercalary, and after a period of 24 h, in the remaining colonies.

A defensive behavior index (DB index) was developed in the present study based on a simple linear model. This index summarizes the weighted values of each defensive behavior variable. In the formula described below, each behavior score (previously registered using the traditional approach described by 86) was multiplied by a fixed numerical value according to its importance for the honey bee hive manipulation by the beekeeper, as follows: 1) The “sting” behavior was considered the greatest impact on the hive management and was, therefore, assigned the highest fixed value per unit (0.15), followed by “fly” (0.05) and “hang” and “run” (0.025) (59).

The DB index values ranged from 0.25 to 1 and were obtained from the following formula:


Index DB=(0.15 x “sting” score)+(0.05 x “fly” score)+(0.025 x “hang” score)+(0.025 x “run” score).

The honey bee colonies were surveyed for the above-mentioned parameters (except phoretic Varroa) six times during the 2019–2021 productive beekeeping season as follows: at the beginning of the beekeeping season during the late spring-summer (December) 2019 (B); autumn (April) 2020 (A); winter (Jun) 2020 (W); early spring (September) 2020 (ES); first Productive Peak (PP1) in spring/summer (December) 2020; and second Productive Peak (PP2) in summer (February) 2021 (**Figure 1**). The colony surveys were performed during each time of the beekeeping season at peak activity hours of the honey bees (between 10 and 12 am) during sunny days with favorable weather conditions (temperature >20°C). For each survey, defensive behavior and strength category were measured on the same day, the behavioral evaluation was performed first and then the category of the colony.

### 2.4 Statistical analysis

Colony strength values were compared among daughter (LE2-LE9) and mother (LE0) colonies in the Tafí Viejo apiary at different times of the beekeeping season (B, A, W, ES, PP1 and PP2) using the Kruskal-Wallis (K-W) test. Brood population data was analyzed by a one-way analysis of variance (ANOVA) (factors = Hive [LE] and Time [B, A, W, ES, PP1 and PP2]).

Defensive behavior variables (run, hang, sting, and fly and the CD index) were compared among colonies at different times of the beekeeping season (B, A, W, ES, PP1 and PP2). All analyses were performed using Kruskal-Wallis (K-W) tests. In addition, a multivariate Principal Coordinate Analysis (PCoA) was performed using Gower’s similarity coefficient (89), based on the defensive behavior variables which resulted statistically significant in the above-mentioned analyses and registered at the ES (early spring) time. This specific moment in the beekeeping season is important to determine the defensive behavior of a colony since it is the season when the colony is leaving the wintering and reinitiating the productive stage. Statistical analyses were performed using InfoStat 2016 (90).

### 2.5 Selection of daughter colonies for NGS analysis

Taking into account the population and behavior parameters registered during the evaluation, the mother (LE0) and two daughter colonies were selected to perform Double Digest Restriction-Site Associated DNA (ddRADseq) according to the following criteria: 1) Stable colony strength parameters during early spring [ES] and productive peaks [PP1 and PP2]), 2) Stable brood population (N° frames with brood >6; % brood/Frame >60) during ES, PP1 and PP2 and, 3) Contrasting and even defensive behavior (most contrasting average values of CD index throughout ES, PP1, PP2) (**Figure 1**). We have selected colonies that presented even defensive behavior during ES-PP1-PP2, since these are the moments when the most intense productive management takes place and the number of individuals within the colony and the volume of entered food are at maximum values.

With this strategy (selected daughter colonies originated from the same mother colony) we expect to detect phenotypic and genetic differences possibly attributed to the differential paternal origin, according to the polyandry of the species (91, 92). Twenty newly-emerged worker bees were randomly chosen and extracted from a brood frame of each colony under laboratory conditions. Each worker was individually placed in 1.5 ml tubes, frozen in liquid nitrogen for 1 min and then preserved frozen (-80°C) to be processed for NGS analyses.

### 2.6 DNA isolation

An individual DNA extraction from each preserved worker (whole body) was performed using a modified CTAB Chloroform/octanol protocol (93). First, grinding was performed with liquid nitrogen to powder. The powder was then transferred to a 1.5 ml tube with 900 μl of extraction buffer (1.4 M NaCl, 20 mM EDTA, 100 mM Tris-HCl and sterile H_2_0). Tubes were incubated at 65°C for 30 min, maintaining gentle agitation. Then 450 μl of chloroform:isoamyl alcohol (24:1) was added and mixing by inversion was performed for 10 min. The resulting emulsions were centrifuged at 14000 rpm for 30 min. The aqueous phase of each sample was recovered into a clean tube, 5 μl of RNase (10 mg/ml) was added, and the tube was incubated at 37°C for 60 min. Then 450 μl of chloroform:isoamyl alcohol (24:1) solution was added, and the samples were mixed by inversion again. The samples were centrifuged again at 14000 rpm for 30 min to recover the aqueous phase of each sample. Finally, the DNA was precipitated by addition of 600 μl of cold isopropanol (-20°C) and subsequent mixing by inversion. DNA precipitates were recovered by spin centrifugation, the supernatant was discarded, and the pellet was washed with 500 μl of cold 70% ETOH (-20°C). After drying, the precipitates were resuspended in 50 μl of sterile H_2_0 (HPLC quality).

The quality of the obtained DNA samples was assessed by electrophoresis in 0.8% w/v agarose gel with GelRed (Biotium) according to the manufacturer’s specifications. Then, DNA samples were sent to the Genomic Unit at IABIMO-CONICET INTA Castelar, Buenos Aires (Argentina), where DNA concentration was measured using Qubit (ThermoFisher). Then, two pools per colony (LE0, LE2 and LE6) were performed with equal amounts of DNA from eight individuals each, yielding a total of six pooled samples (16 workers from each colony).

### 2.7 Preparation and sequencing of ddRADseq libraries

Double Digest Restriction-Site Associated DNA (ddRADseq) requires a particular combination of two restriction enzymes in the digestion step (63, 67). Regarding the criteria for selecting the enzymes for the *A. mellifera* genome digestion, EcoRI and MspI had been previously tested with other enzymes involving the ddRADseq technique (68, 71, 73), whereas MboI had been used in mitochondrial DNA studies of the genus *Apis* (94) and mapping approaches (95, 96). In the present study, two pairs of restriction enzymes were tested for their usage in the digestion step before ddRADseq library construction: the *Msp*I/*Eco*RI combination, selected according to previous bibliography (73), and the *Mbo*I/*Eco*RI pair, available from the sequencing service (Genomic Unit at IABIMO-CONICET INTA, Argentina). In silico digestion was carried out using the reference genome of *A. mellifera* Amel_HAv3.1 (NCBI Genome Assembly) and SimRAD package (97) in R. In addition, this test of restriction enzymes was performed using a set of randomly selected DNA samples (obtained in this study) to confirm the size and quantity of the resulting fragments. Pooled DNA samples were processed, and ddRADseq libraries were constructed following the protocol described by Aguirre et al. (98) using the *Mbo*I-*Eco*RI combination in the digestion step. Paired-end sequencing (2x250) was performed on a Novaseq6000 (SAGA-CIMMYT, Mexico).

### 2.8 Bioinformatic analysis

First, the quality of the batches was checked visually by using FastQC (99). Sequences were filtered by quality using the “process_radtags” program from the Stacks v2.62 package (100) with default parameters. Adapters and poor-quality sequences were removed using Trimmomatic v0.32 (101) with recommended settings. The clean reads were mapped to the reference genome of *A. mellifera* from NCBI Genome Assembly (Amel_HAv3.1), using Bowtie2 v2.4.5 (102) under default settings by first indexing the genome and then mapping the ddRADseq short reads. To assemble loci according to the alignment positions provided for each read and SNP calling, we ran the “ref_map.pl” program (Stacks). The “populations” program was run afterward to generate population-level summary statistics; the corresponding raw SNP matrix was obtained under Variant Call Format (VCF).

To predict the functional effect of polymorphisms, the Variant Effect Predictor (VEP) from the Ensembl Metazoa (version release 55) (103) was used. The density of SNPs per chromosome was plotted using CMplot package (v.3.1.3) (104) in R (105, 106).

For pathway mapping and functional enrichment analysis, ShinyGO v0.76.3 software (107) was used with default settings, including: pathway database for gene count option was allowed; statistically significant pathways were selected by FDR and sorted by Fold Enrichment. The g:Profiler ve107_eg54_p17_bf42210 was also used with database updated on 15/09/2022 (108). The in-cis potential targets of the SNPs annotated as parts of lncRNAs were searched. Protein-coding genes 10 k to 100 k upstream and downstream of lncRNAs were identified and their function was further investigated using KOBAS (version 3.0) (109) and the NCBI database.

A dendrogram was inferred using the filtered SNPs among the six ddRADseq libraries from the VCF file, based on pairwise identity-by-state (IBS) values using the SNPRelate package version 1.32.2 (110). The dendrogram was constructed using the maximum likelihood hierarchical clustering analysis implemented in the snpgdsHCluster (SNPRelate) option and plotted with the R package ggplot2 (111). Chi-square analyses (112) were performed to visualize the genetic differences between colonies (LE2, LE6 and LE0) in relation to population parameters, namely heterozygosity (HTZ), reference homozygosity (REF HMZ) and alternative homozygosity (ALT HMZ), using InfoStat 2016 software (90).

## 3 Results

### 3.1 Colony status analysis

**Figure 2:**
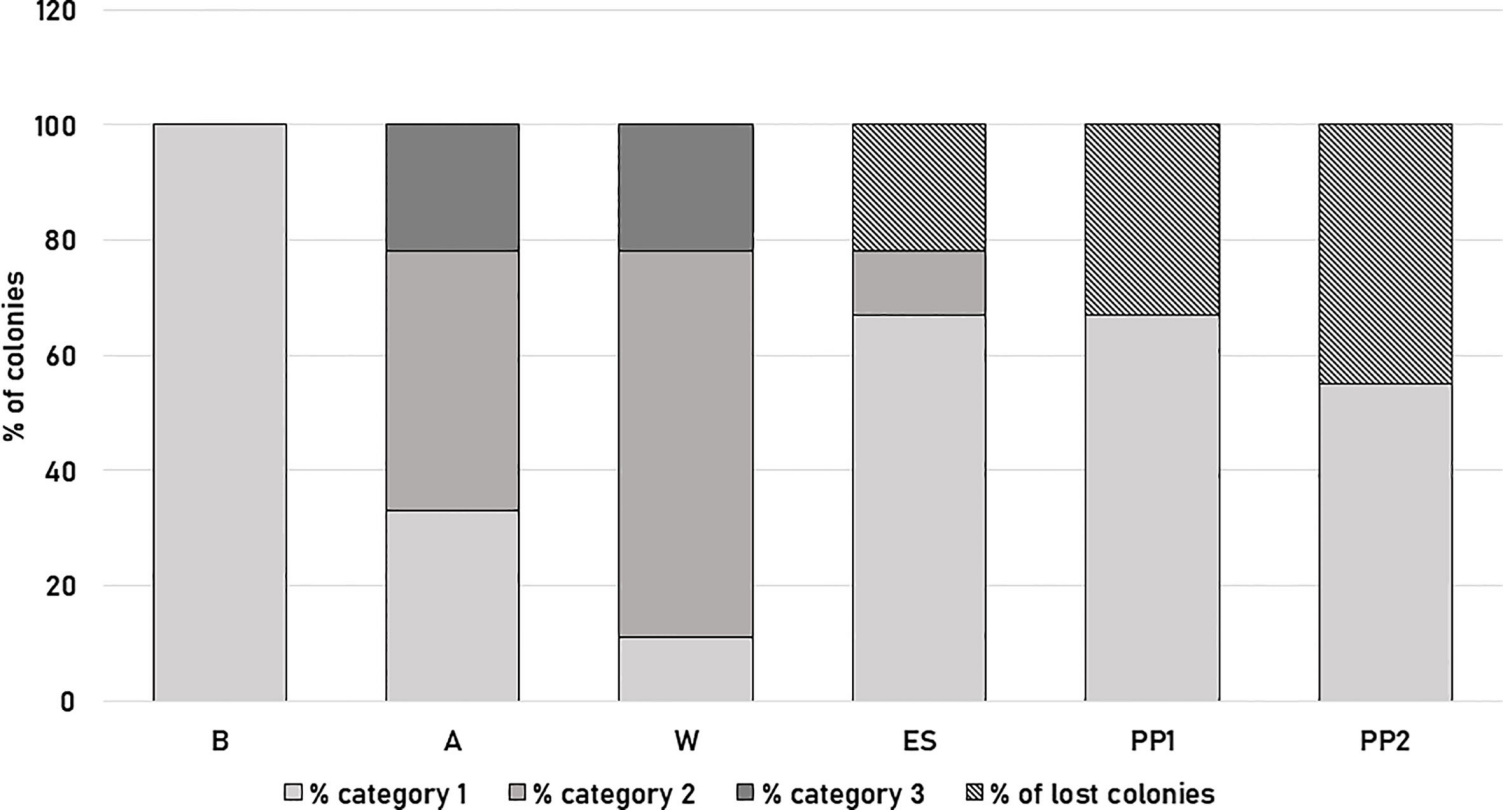
Colony strength of *A mellifera* colonies from Tafí Viejo apiary at six specific times during the beekeeping season: late spring-summer 2019 (B), autumn 2020 (A), winter 2020 (W), early spring 2020 (ES), 1st productive peak 2020 (PP1) and 2nd productive peak 2020 (PP2). In different colors, the mean percentage of colonies in category 1 (at least seven frames covered by worker bees), category 2 (five to seven frames covered by worker bees) or 3 (four or fewer frames covered by worker bees) and percentage of lost colonies are shown.

All colonies surveyed showed the greatest strength at the beginning of the survey (category 1, **Figure 2**; **Supplementary Table 1**). In autumn, two daughters (LE6, 7) and the mother (LE0) colonies (33%) remained in category 1, while 45% of the colonies (four daughter colonies LE2, 4, 8 and 9) dropped to category 2, and 22% (LE1 and LE3 changed drastically to category 3 (**Figure 2**). During winter, 67% of the colonies (six daughter colonies LE1, 2, 4, 6, 7 and 8) were in category 2, 22% (2 colonies, LE3 and LE9) in category 3 and only the mother colony (LE0) was in category 1 (**Figure 2**). In early spring, two colonies (LE1 and LE3) (22%) were considered lost, one colony (LE8) remained in category 2 (11%), and six colonies (the mother colony and five daughters LE2, 4, 6, 7. 8, and 9) were upgraded to category 1 (67%) (**Figure 2**). During the first productive peak (PP1), 33% (three daughter colonies LE1, 3 and 8) recorded losses and the remaining six colonies (LE2, 4, 6, 7, 9 and the mother colony LE0) were in category 1 (67%). For the second productive peak (PP2) we registered five colonies (daughter LE2, 6, 7, 9 and the mother (LE0) colonies; 55%) in category 1 and 45% of the colonies (daughter colonies LE1, 3, 4, 8) were registered as lost (**Figure 2**). No significant differences were found for colony strength between colonies (H= 2.04, p=0.96 K-W test). However, a statistically significant difference was observed between the six moments of the beekeeping season evaluated (B, A, W, ES, PP1 and PP2) (H= 25.39, p= <0.0001 K-W test).

The brood population of the surveyed colonies showed dissimilar patterns throughout the beekeeping season for both the number of brood frames and the percentage of brood per frame, except in early spring. During this time of the season, we observed convergent dynamics of the different colonies in terms of the number of brood frames (**Supplementary Table 1**). In addition, significant differences were observed for the number of fully covered frames per brood (F=5.83; p<0.0001) and the percentage of brood per frame (F=2.56; p=0.0217) among the colonies. However, non-significant differences were observed among colonies for times of the year. Regarding phoretic Varroa, we observed a high variability among the obtained values for each of the surviving colonies at the time of sampling (ES), with a mean of 4.55% and a deviation equal to 3.89 (**Supplementary Table 1**).

### 3.2 Defensive behavior analysis

**Figure 3:**
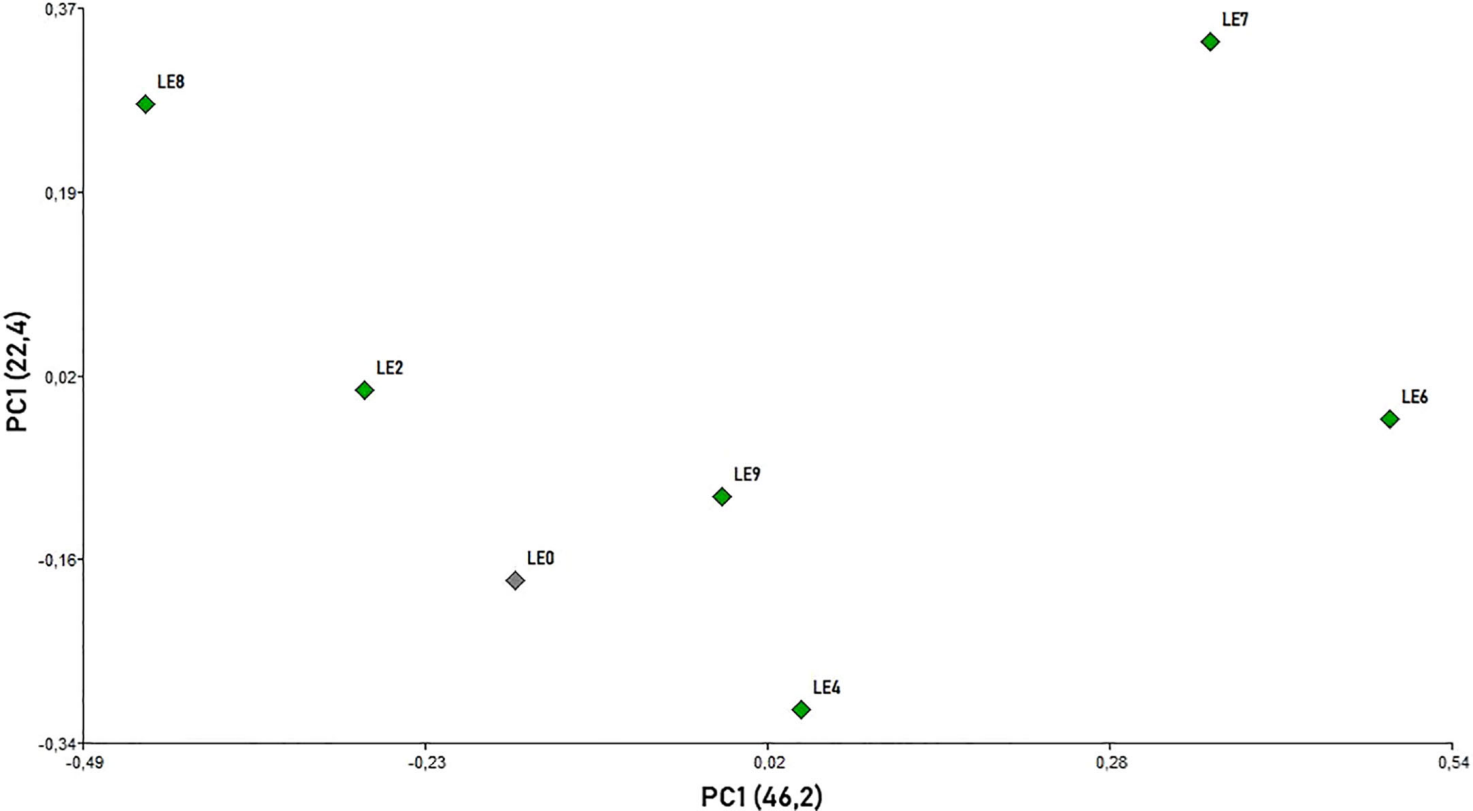
Principal coordinate analysis (PCoA) scatterplot for *A. mellifera* colonies based on all defensive behavior variables using Gower’s similarity coefficient (89).

The analysis of defensive behavior variables (Kruskal-Wallis test) showed significant differences between the mother and daughter colonies for “run” (H=16.00; p=0.0163), “hang” (H=14.76; p= 0.0056), “sting” (H= 18.28; p= 0.0026) and the DB Index (H= 15.16; p=0.0483). However, no significant differences were observed between colonies for “fly” (H= 9.44; p=0.2115). In addition, the same dynamics of defensive behavior was observed between the mother and daughter colonies among the different evaluation times during the beekeeping season (“run” [H=3.14; p=0.5958], “hang” [H=0.68; p= 0.9627], “sting” [H= 4.30; p= 0.3511], DB Index [H= 6.75; p=0.2242] and “fly” [H= 6.78; p=0.1694]) (**Supplementary Table 1**). The distribution of evaluated honey bee colonies in the two-dimension space (PCoA) confirmed that the behavior variables exhibiting significant differences (run, sting, hang and DB Index) were useful to explain 68.6% of the observed variability in the first two components (PC1 = 46.2; PC2 = 22.4; **Figure 3**). LE2 and LE6/LE8 and LE7 were located in opposite positions in the X-axis, while LE4 and LE9 and the mother colony (LE0) were centrally located (**Figure 3**).

### 3.3 Selection of daughter colonies and sample preparation for NGS analysis

Two daughter (LE2 and LE6) and the mother (LE0) colonies were selected for NGS assay according to the results registered for their biological and behavior parameters, as follows: the three colonies maintained a stable colony strength (category 1) and stable brood population (N° frames with brood >6; % brood/Frame >60) throughout the ES, PP1 and PP2 time period. Also, the two daughter colonies (LE2 and LE6) displayed contrasting and stable defensive behavior. Specifically, we observed the lowest average CD index (0.31) in the LE2 daughter colony and the highest average CD index (0.70) in the LE6, while the mother colony (LE0) showed a mean value (0.39) throughout the time period (**Supplementary Table 1**). The in silico enzymatic digestion of the reference DNA (*A. mellifera* Genome Assembly, Amel_HAv3.1 NCBI) with *Mbo*I/*Eco*RI combination yielded a higher number of fragments (16,035 fragments) of the desired size (~450 to 500 bp) than *Msp*I/*Eco*RI combination (7,562 fragments). The number of total cut sites in the reference genome was 239,138 for *Msp*I, 63,098 for *Eco*RI and 61,422 for *Mbo*I, hence demonstrating a higher efficiency of the first enzyme combination for the preparation of NGS samples. In addition, both enzyme combinations tested in the laboratory confirmed the in silico results (results not shown).

### 3.4 ddRADseq analysis

The analysis of the six ddRADseq libraries (LE0, LE2 and LE6; two replicates each) yielded a total of 16,360,032 raw paired-end reads (2x250). After cleaning the raw data, a mean value of 99.4% of reads was retained (**Supplementary Table 2**). An average alignment rate of 60.5% was obtained after mapping reads to the reference genome. A total of 12,971 variant sites (SNPs) were found to be distributed in 33,053 assembled loci.

**Figure 4:**
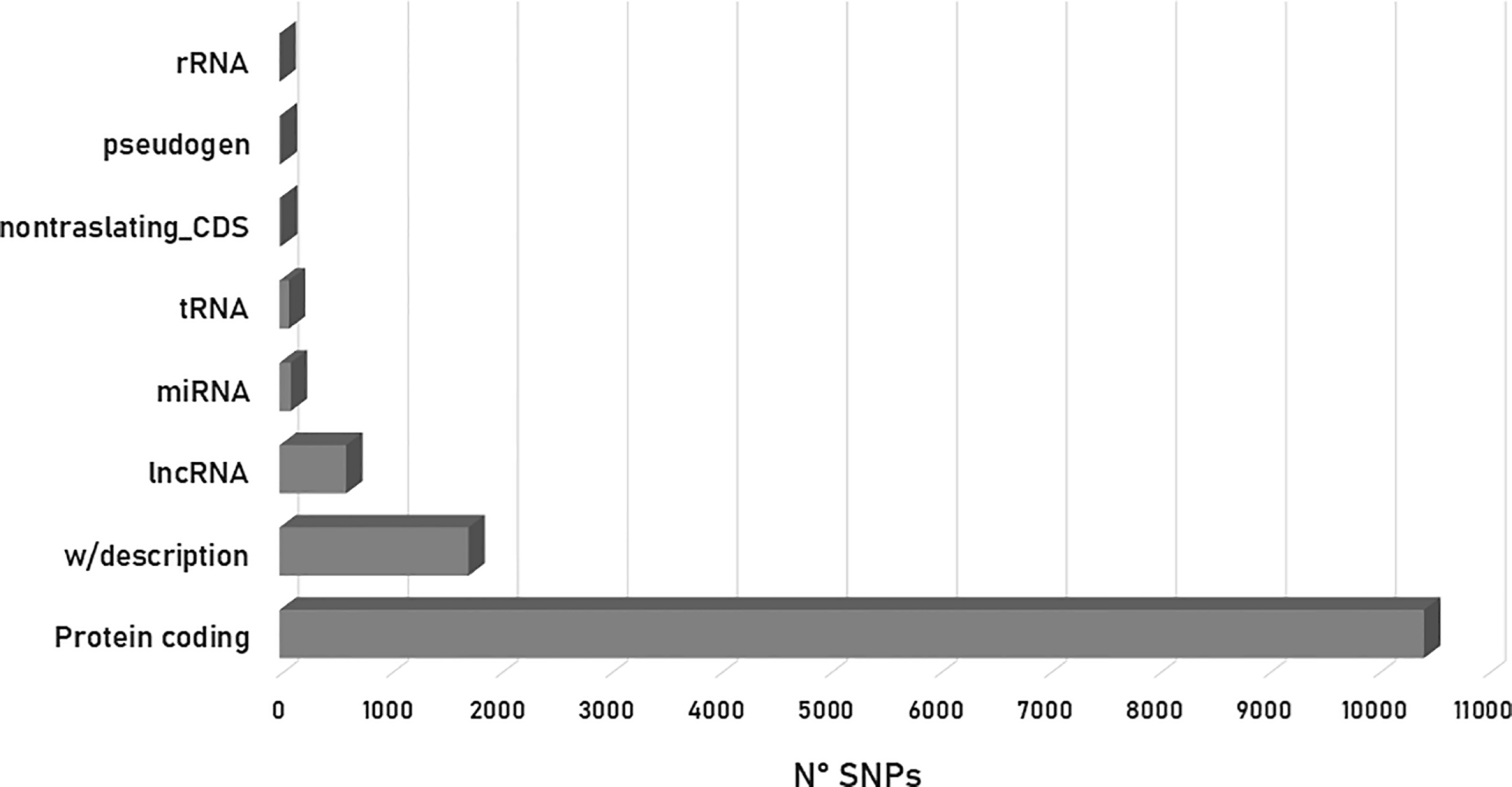
Histogram depicting the number of SNPs associated with biological effects according to VEP (Ensembl Metazoa).

Of the 12,971 variant sites processed, 80.40% (10,492 SNPs) were identified as present in protein-coding regions. From this percentage, 73% had a synonymous effect whereas 26% was predicted to cause changes in the amino acid sequence (missense variants). The remaining 13.27% (1,721 SNPs) was cataloged as without description, 4.68% (607 SNPs) as lncRNA, 0.79% (103 SNPs) as miRNA, 0.11% (14 SNPs) as non-translating CDS, 0.08% (10 SNPs) as pseudogenes and 0.66% (86 SNPs) and 0.01% (1 SNP) as tRNA and rRNA, respectively (**Figure 4**). In addition, 70% of the SNPs that produced a variant effect corresponded to intronic variants, accounting for more than half of the variants detected, while upstream and downstream gene variants accounted for 9% and 7%, respectively (**Figure 4**). The remaining percentage values were distributed as follows: non-coding transcript variant (5%), intergenic variant (3%), synonymous variant (2%), 3-prime UTR variant (1%), 5-prime UTR variant (1%) and others (3%).

The functional annotation of 2,527 genes and subsequent gene enrichment analysis identified 113 GO terms. These terms were classified into functional categories such as cellular component (CC, 12 GO terms), molecular function (MF, 30 GO terms) and biological process (BP, 70 GO terms), as well as KEGGs (Kyoto Encyclopedia of Genes and Genomes) pathways (1 term) (**Supplementary Table 3**). Enriched GO terms for CC category included plasma membrane, cell periphery, cell junction, intrinsic component of plasma membrane and synapse. The most significantly enriched GO terms for the MF category were associated with DNA-binding transcription factor, transcription regulator, calcium ion binding and G-protein-coupled receptor activities. GO terms enriched in BP were mainly related to the regulation of biological processes, such as nucleic-acid transcription, biosynthesis, signaling, cell communication and developmental processes (See **Supplementary Table 3**). The neuroactive ligand-receptor interaction category from the KEGG database was shown to be enriched with 31 genes belonging to this term (**Supplementary Figure 1**).

**Figure 5:**
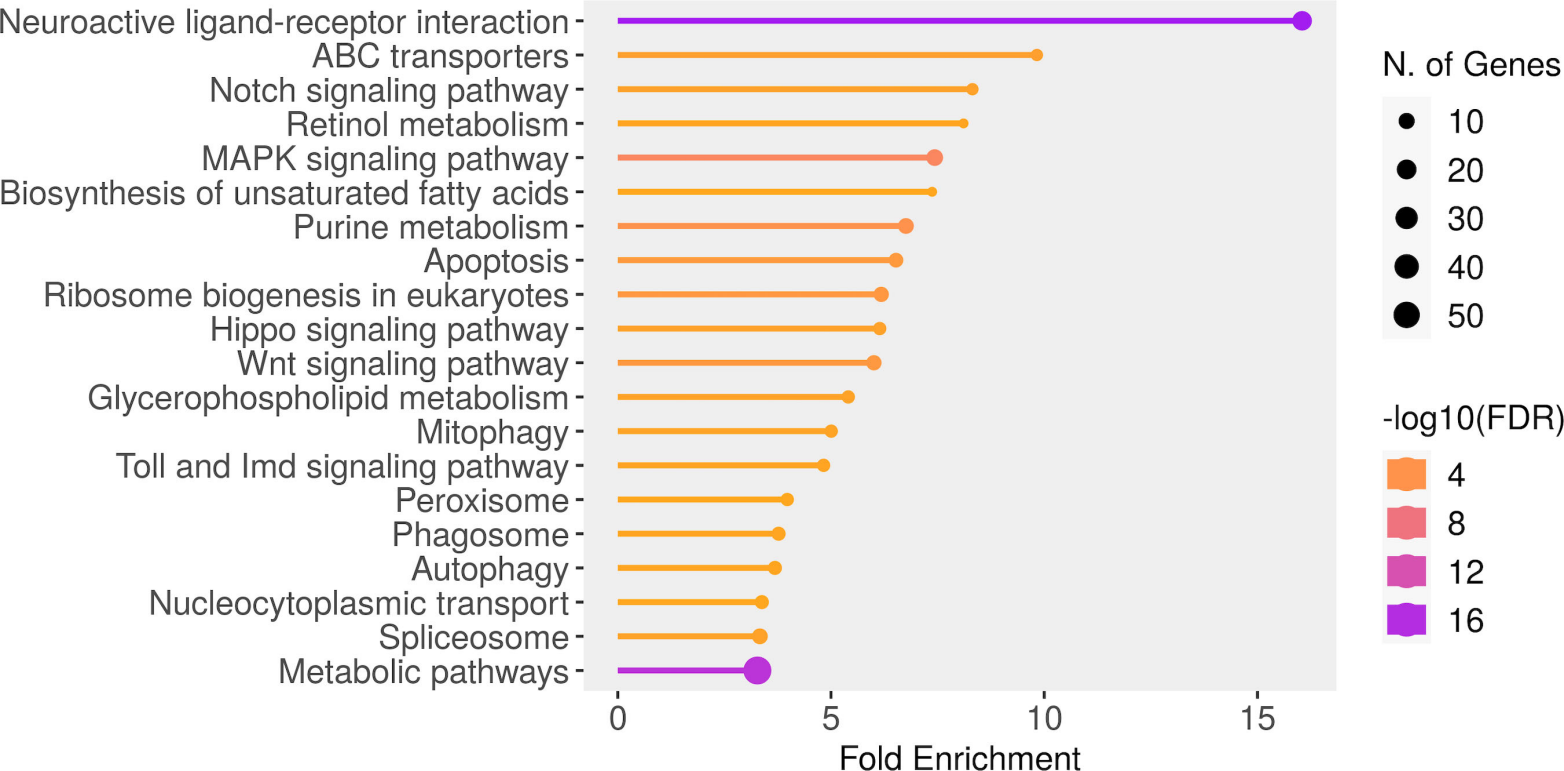
Significant molecular pathways selected by FDR and sorted by Fold Enrichment generated (ShinyGo tool).

A total of 20 pathways yielded an FDR value of less than 0.035 (**Figure 5**). The most enriched pathways included “Neuroactive ligand-receptor interaction” (19 genes), “ABC transporters” (4 genes), “Notch signaling pathway” (4 genes) and “Retinol metabolism” (3 genes). Among the top 20 enriched pathways, “Metabolic pathways”, “Neuroactive ligand-receptor interaction” and “MAPK signaling” together included most of the genes of the data (**Figure 5**). The SNPs detected in the 5-HT genes are part of the neuroactive ligand-receptor interaction pathway. The identified variants are mostly intronic polymorphisms, potentially involved in the alternative splicing by interfering with splice site recognition (**Supplementary Table 4**).

**Figure 6:**
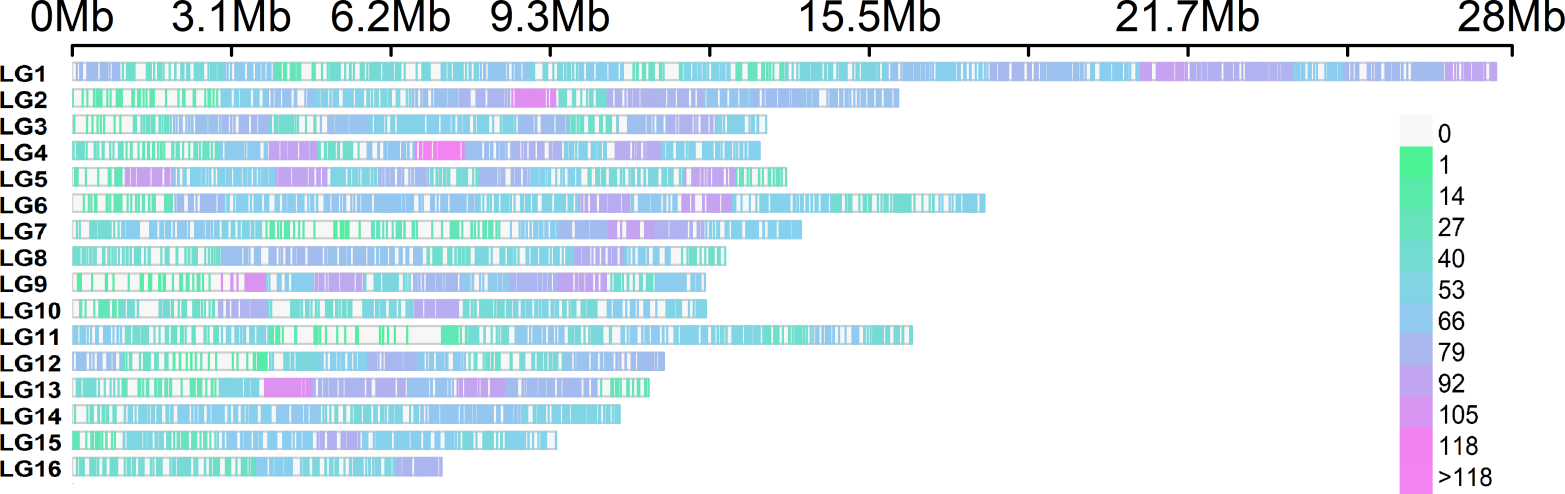
SNP density across the 16 chromosomes (linkage groups) of *A. mellifera* representing the number of SNPs within 1 Mb window size. The horizontal axis represents the chromosome length in Mb. Different colors correspond to SNP density (color scale on the right side of the image). LG, linkage groups.

**Figure 7:**
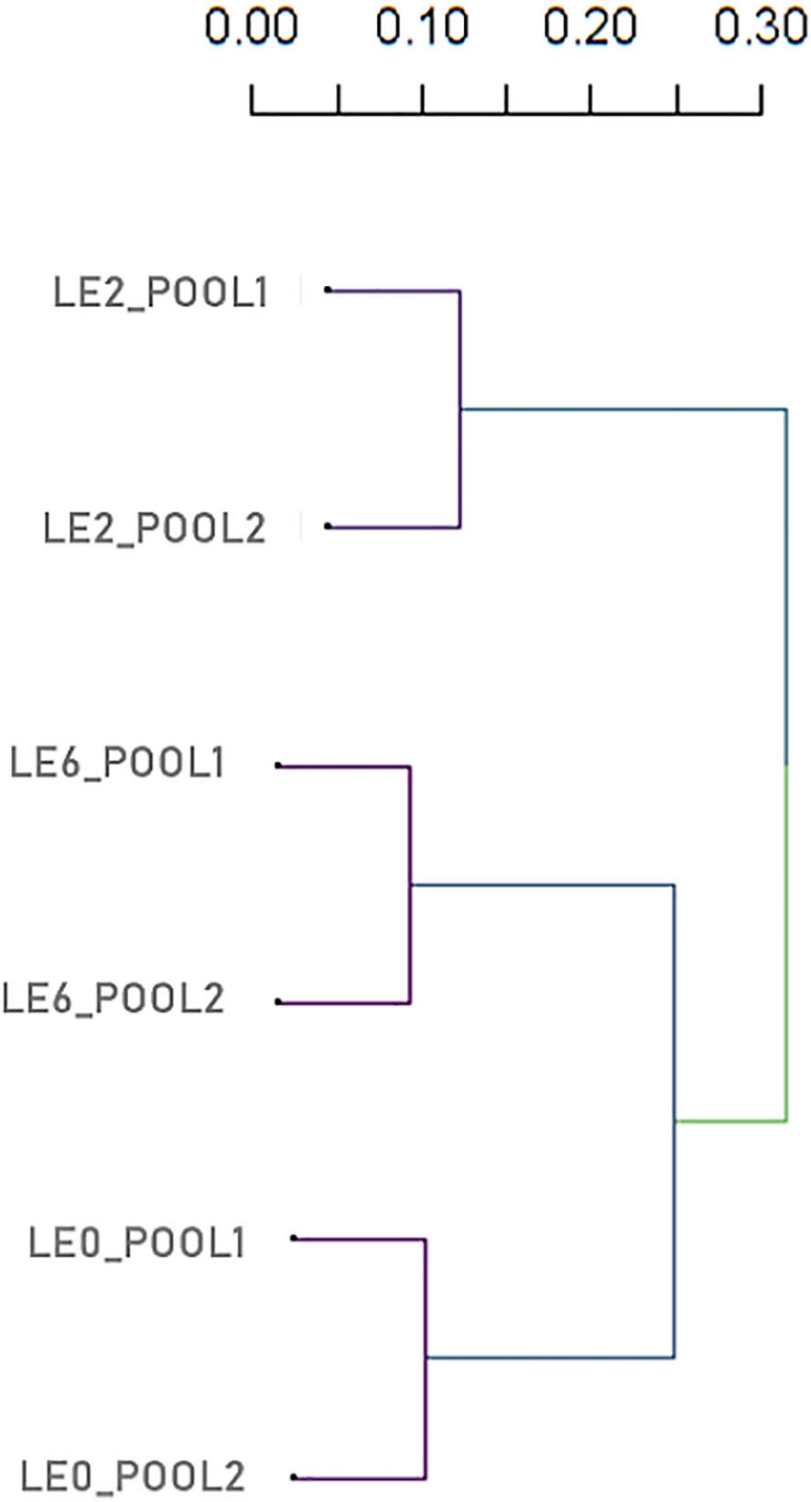
Dendrogram based on hierarchical cluster analysis (pairwise identity-by-state values) from SNP data of six *A. mellifera* colonies.

The graphical representation of the SNP distribution (12,971) along the *A. mellifera* reference genome demonstrated the presence of SNPs in the 16 linkage groups, which was enhanced in LG1 (**Figure 6**). The statistical analysis of the SNP matrix revealed 142 SNPs differentially present between samples (LE0, LE2 and LE6). Hierarchical cluster analysis of the matrix displayed in a dendrogram (**Figure 7**; **Supplementary Table 5**) showed a clear clustering of the samples, with LE0 being more closely related to LE6, which exhibited a higher level of defensive behavior than LE2.

From a total of 12,971 polymorphic sites, 647 SNPs were located in protein-coding regions corresponding to 128 genes with mostly known functions. Eighty-two percent of them were annotated as related to different aspects of behavior, namely development, morphology, immune response and caste division, among others. From this SNP set, 128 SNPs were mapped in 21 genes previously reported to be involved in defensive behavior, including *dop3* and *dopR2*, *Camkii* and *Adar*, *obp9* and *obp10* or members of the 5-*HT* family (**Supplementary Table 6**).

**Figure 8:**
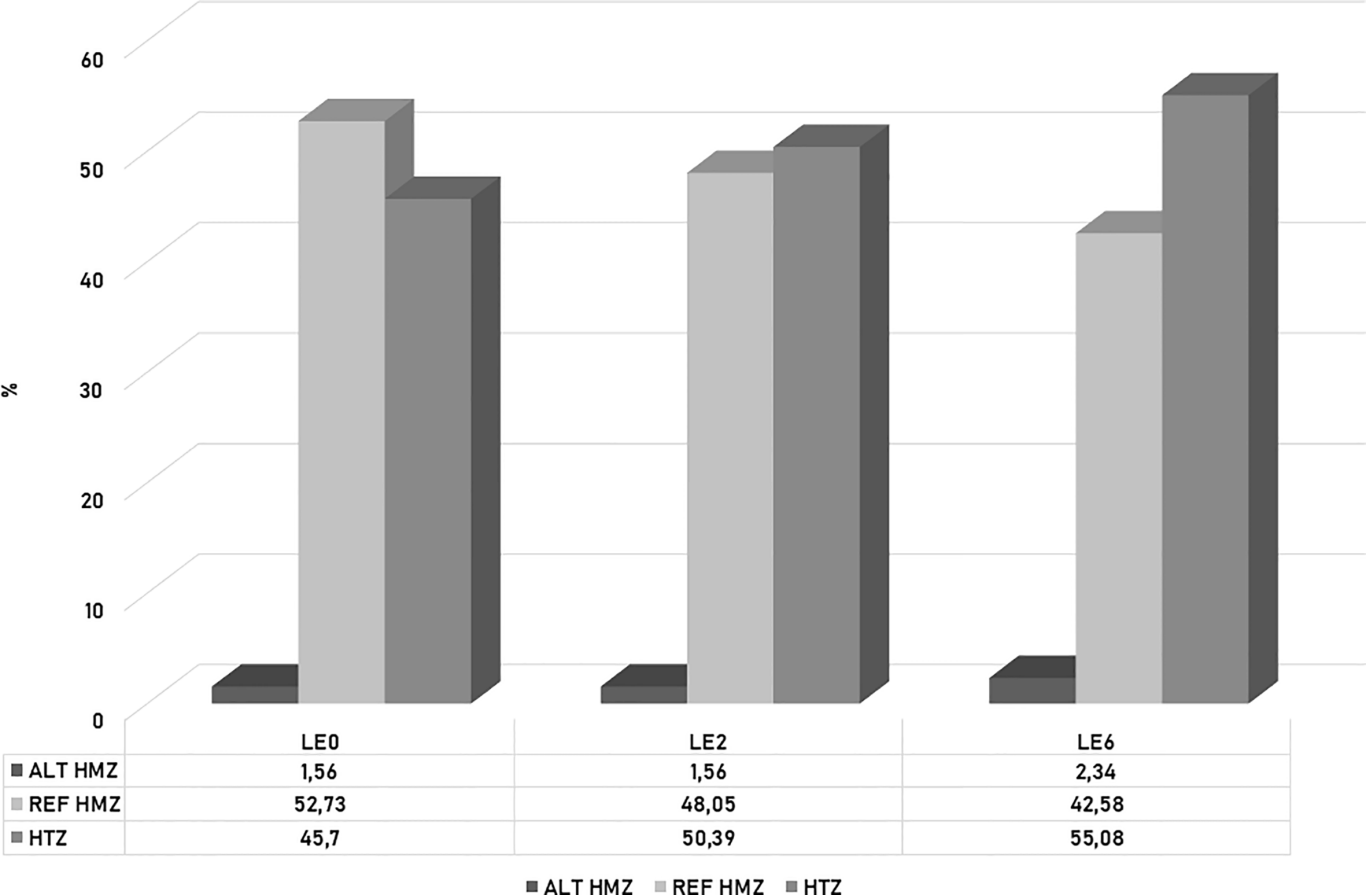
Histogram of the percentage of heterozygosity (HTZ), reference homozygosity (REF HMZ) and alternative homozygosity (ALT HMZ) observed for 128 SNPs associated with defensive behavior in the *A. mellifera* colonies analyzed.

A population analysis of this set of 128 SNPs revealed that LE0 had the highest percentage of reference homozygosity (REF HMZ) (52.63%) and the lowest percentage of heterozygosity (HTZ) (45.7%). LE6 showed the highest percentage of heterozygosity (55.08%), as well as alternative homozygosity (ALT HMZ) (2.34%) and the lowest percentage of REF HMZ (42.58%). In contrast, LE2 exhibited the lowest value for ALT HMZ (1.56%), together with LE0 (**Figure 8**). At the statistical level, significant differences were observed between samples (Chi-square Pearson: χ2 = 25.4, p = 0.0046) but not between colonies (χ2 = 5.57, p = 0.2334).

We further investigated the possible role of the lncRNAs, in which 607 SNPs were detected by predicting the potential targets of lncRNAs by in-cis regulation. A total of 1,232 target loci between 10 and 100 kb up and downstream from 178 lncRNAs were predicted in the in-cis role. Among them, 28 genes and 18 microRNAs were included (**Supplementary Table 7**). Some of these genes included *Nxr-1*, *NLG-5*, *Dop3*, *Dnmt1a*, *Y-y*, *Silk Fibroin 1 2 3* and *4*, *E75*, *nAChRb1*, *Pla2*, *mcdp* and *apamin*, among others. Using KOBAS enrichment module, we identified three significantly enriched pathways (p-value<0.05) in the predicted in-cis targets of the lncRNAs. The pathways were related to metabolism, such as alpha-linolenic acid metabolism, oxidative phosphorylation, and retinol metabolism.

## 4 Discussion

The characterization of *A. mellifera* ecotypes adapted to subtropical climate and the understanding of their genotypic and phenotypic diversity are essential for efficient breeding, conservation, and management of honey bee germplasm. In the present study, we developed a protocol for the selection of Africanized honey bee stocks based on desirable phenotypic characteristics for beekeeping (colony strength, brood population and defensive behavior) and analyzed the genetic variability of colonies that displayed contrasting defensive behavior as a first approach to understanding possible molecular pathways associated with this behavior in the analyzed honey bee stock.

The results showed homogeneity in the adult population (strength) among the surveyed colonies throughout the beekeeping season. This finding indicates that the mother colony and its daughters had similar dynamics in terms of the number of adult worker bees. However, we found significant differences among colonies at different times of the beekeeping season. These differences were mostly attributed to the difference in the sensitivity of the colony to changes in the environment and to the natural population dynamics of colonies in subtropical climate (58, 113–115) performed by honey bees from each colony during the time surveyed. In addition, we observed significant differences between colonies for both variables of brood population, the percentage of brood per frame and the number of frames with brood. For the percentage of brood per frame, the greatest differences were observed during autumn and early spring, which could be related to the differential response of each colony to the changing climatic conditions and the internal management of colony resources (113, 114). Likewise, the variation registered in the number of brood frames during early spring and the first productive peak could be linked to a differential response to the activation of the natural food input to the colony (33). The reported colony losses in our study might be related to several factors depending on the seasonality. For instance, during early spring, the loss of Africanized colonies is associated with a more marked tendency to swarm due to the reentry of food into the colony and the subsequent explosive population growth (9, 116). During productive peaks, losses are mainly related to avoidance behavior, also highly frequent in Africanized colonies. This behavior is described as a consequence of environmental stressors such as handling, the presence of harmful particles in food sources and excessive rainfall, among others (117).

The experimental design used in the present study, including the analysis of behavior, population, and genetic variables from mother and daughter *A. mellifera* colonies, revealed that the variability reported for four of the five defensive behavior parameters are potentially associated with genetic differences of paternal origin. Firstly, the environmental factors and beekeeping practices must have affected the colonies in the same way, as they shared location and management. Secondly, according to previous studies (38, 48, 82, 118), defensive behavior displays characteristics of dominance and paternal bias, mainly related to the variability generated by the mating behavior and reproductive system of *A. mellifera* (reproduction managed by a single fertile female mated by 12 to up to 30 drones; (119, 120). The paternal influence on this character could explain the statistical differences detected in our study between the LE0 colony and the daughter colonies since they share the same maternal background.

The DB (Defensive Behavior) index developed in this study to summarize the defensiveness displayed by the *A. mellifera* colonies surveyed was consistent with the unweighted values of each of its components. In addition, this index was useful as a selection criterion to detect the two daughter colonies (LE2 and LE6) which exhibited contrasting defensive behavior for ddRADseq analysis. Further surveys under field conditions are needed to test its usefulness as a potential tool for the selection of *A. mellifera* colonies by low defensive behavior under a subtropical climate.

The ddRADseq technique utilized in the present study set up a new combination of restriction enzymes (*Mbo*I/*Eco*RI) yielding a higher number of DNA fragments of desired size (~450 to 500 bp) than the other enzyme combination (*Msp*I/*Eco*RI) tested. *Eco*RI and *Msp*I have been previously tested in *A. mellifera*, in conjunction with other enzymes involving ddRADseq studies (68, 71, 73). Therefore, the present study provides a precedent for the use of *Mbo*I/*Eco*RI for ddRADseq analysis of Africanized *A. mellifera* colonies.

Regarding ddRADseq libraries, even though sequence retention after quality filtering was very high, we obtained both a lower alignment rate against the Amel_HAv3.1 reference genome and fewer SNPs between samples in comparison with previous studies (68, 71, 72). We expected these results because we used hybrid genetic material instead of pure subspecies, as shown in the cited studies. These differences can also be attributed to the sample size and the fact that our samples were closely related to each other, being derived from the same mother colony. Further studies with a higher number of colonies should be performed to explore in greater depth the genotypic differences detected.

We identified a total of 12,971 SNPs in the overall comparison of mapped reads against the reference *A. mellifera* genome distributed in the 16 linkage groups (chromosomes), showing a wide SNP coverage in all linkage groups. In 142 SNPs, we found significant differences between paired comparisons of the analyzed samples. According to the distance matrix generated based on this SNP set, the least defensive daughter (colony LE2) proved to be the most distant from the mother colony, LE0, which was relatively closer to the colony that exhibited the highest defensiveness (LE6). These results made evident the paternal influence on this character and the need for controlled conditions to minimize the defensive behavior in daughter colonies, as previously reported by Giray et al. (46), Büchler et al. (74), Lenoir et al. (121).

The analysis of mapped SNPs in protein-coding regions, evidenced the presence of this type of polymorphisms in 21 genes putatively related to defensive behavior according to previous studies, as modulators of learning and memory, olfactory receptors, neurotransmitters or components of both the nervous and immune systems. Some of these genes were as follows: *dop3* and *dopR2*, from the dopamine family, associated with learning and memory (122); *Camkii* and *Adar*, identified as behavior modulators by Whitfield et al. (123); *obp9* and *obp10* belonging to the OBP family, involved in olfactory sensitivity (124); and members of the 5-HT family (four present among the genes detected in the present study) related to neurological processes through the regulation of octopamine and serotonin receptors (125–127). Recently, Acevedo-Gonzalez et al. (128) have described a direct relationship between the 5-HT family genes and the expression of defensive behavior in Africanized honey bees from Puerto Rico, specifically for genes identified as *5HT2a* (alpha and beta). On the whole, the available information should indicate common molecular pathways underlying this behavior in pure and hybrid honey bee lineages.

Notably, in the present study, from the 128 SNP set associated with the defensive response, the most defensive colony, LE6, showed higher alternative homozygosis and heterozygosis than the least defensive daughter colony, LE2, whereas the mother colony had the highest percentage of REF HMZ and the lowest HTZ values. Taking these results into account, we assume that the observed behavioral variation could be closely related to the genetic differences detected by ddRADseq, which have a high paternal influence, in agreement with previous studies (48, 82, 118). The colonies might have different paternal origins, given the polyandry present in the species. These preliminary results need further analysis by surveying a greater number of colonies and generations to support the association between behavior and genetics.

We also detected SNPs in long non-coding RNAs (lncRNAs). Previous studies have confirmed the role of lncRNAs in many biological processes, such as cell differentiation and development, as well as immune responses and tumorigenesis (129). Further analysis of the possible in-cis target regions of the identified lncRNAs performed in the present study revealed that previously studied genes were involved in nervous processes such as Neurexin 1 (*Nrx-1*), Neuroglin 5 (*NLG-5*) (130) and D2-like dopamine receptor (*Dop3*) (131). Other putative target genes include DNA methyltransferase 1a (*Dnmt1a*) (132), yellow-y (*Y-y*) (133), ecdysone-induced protein 75 (*E75*) (134), nicotinic acetylcholine receptor (*nAChRb1*) (135) and bee venom components such as phospholipase A2 (*PLA2*), mast cell degranulating peptide (*mcdp*) and *apamin* (136–138). This raises the possibility that the lncRNAs found in this study with differential SNP frequencies between sister colonies with contrasting behavior may be acting as regulators of the defensive response and opens the door to further investigations to elucidate the role of these lncRNAs in defensive behavior.

## 5 Conclusion

Phenotypic and genetic characteristics of an Africanized *A. mellifera* stock tolerant to *V. destructor* and displaying variability to defensive behavior were evaluated here. Our results suggest a strong paternal influence, evidenced by the polyandry exhibited by this species (multiple fathers contributing to the expression of this character), in agreement with previous findings. The tools and protocols developed and used in the present study, such as the DB index, *Mbo*I/*Eco*RI restriction enzyme combination and ddRADseq, as well as the SNP dataset generated, could be used in future honey bee selection projects based on defensive behavior and adaptability to a subtropical climate. These promising tools would add innovation to honey bee breeding programs at the regional level, thus improving the productive capacity and competitiveness of small and medium-sized honey producers. The selection and multiplication of honey bee colonies with promising characteristics for beekeeping can be benefited by the saturation of drone congregation zones with desired and known genetic characteristics and/or the use/implementation of reproduction stations and the movement of germplasm as have been previously described (3 and references therein).

Our results set a precedent for the use of ddRADseq for the identification of molecular markers and polymorphisms potentially associated with defensive behavior, mainly those related to the 5HT gene family and lncRNA. The gained information paves the way for further research using a larger number of contrasting daughter colonies for the trait of interest in order to perform linkage analyses between this behavior and major genes involved in its expression.

## Data availability statement

The datasets presented in this study can be found at https://www.ncbi.nlm.nih.gov/, accession number: PRJNA1005491.

## Author contributions

EB and SL conceived and designed the study. EB, SL, AS, GR and GG participated in the design of experiments and the establishment of colony selection criteria. EB, GG and GR maintained and selected the *A. mellifera* colonies, EB performed DNA isolation experiments. NA, CVF and CA directed the bioinformatic analyses. EB and CF participated in the bioinformatic analyses. AP and PV processed the DNA samples for sequencing. EB, CF, and SL wrote the manuscript. All the authors read and accepted the manuscript, contributed to the article, and approved the submitted version.

## Supplementary material

Supplementary Figure 1 GO enrichment analysis. Neuroactive ligand-receptor interaction category (KEGG).

Supplementary Table 1 Strength, population and behavior parameters of the analyzed *A. mellifera* colonies. “-” the parameters were not assessed due to the colony loss. The phoretic varroa were measured for the sole purpose of confirming the status of tolerance of the honey bee stock.

Supplementary Table 2 Percentage of reads retained after filtering with the “process_radtags” program from Stacks 2 (100).

Supplementary Table 3 g_GOSt result table containing information about the enriched terms, term sizes and corresponding P-values from gProfiler (108).

Supplementary Table 4 Pathway enrichment analysis generated with the ShinyGo tool (107).

Supplementary Table 5 Genetic distances between ddRADseq samples using filtered SNPs and based on identity-by-state (IBS) values (SNPRelate, R package).

Supplementary Table 6 Annotation, position and population parameters of 128 SNPs mapped in 21 A*. mellifera* genes and previously reported as involved in defensive behavior.

Supplementary Table 7 List of lncRNAs IDs from the total set of SNPs (first sheet). LncRNA putative in-cis targets genes 100 kb upstream and downstream (second sheet).
