# Supplementary figures and images for: Phenotypic and genetic characterization of Africanized Apis mellifera colonies with natural tolerance to Varroa destructor and contrasting defensive behavior
Source: Front Insect Sci. 2023 Aug 31;3:1175760. doi: 10.3389/finsc.2023.1175760 (PMC10926445; doi:10.3389/finsc.2023.1175760)

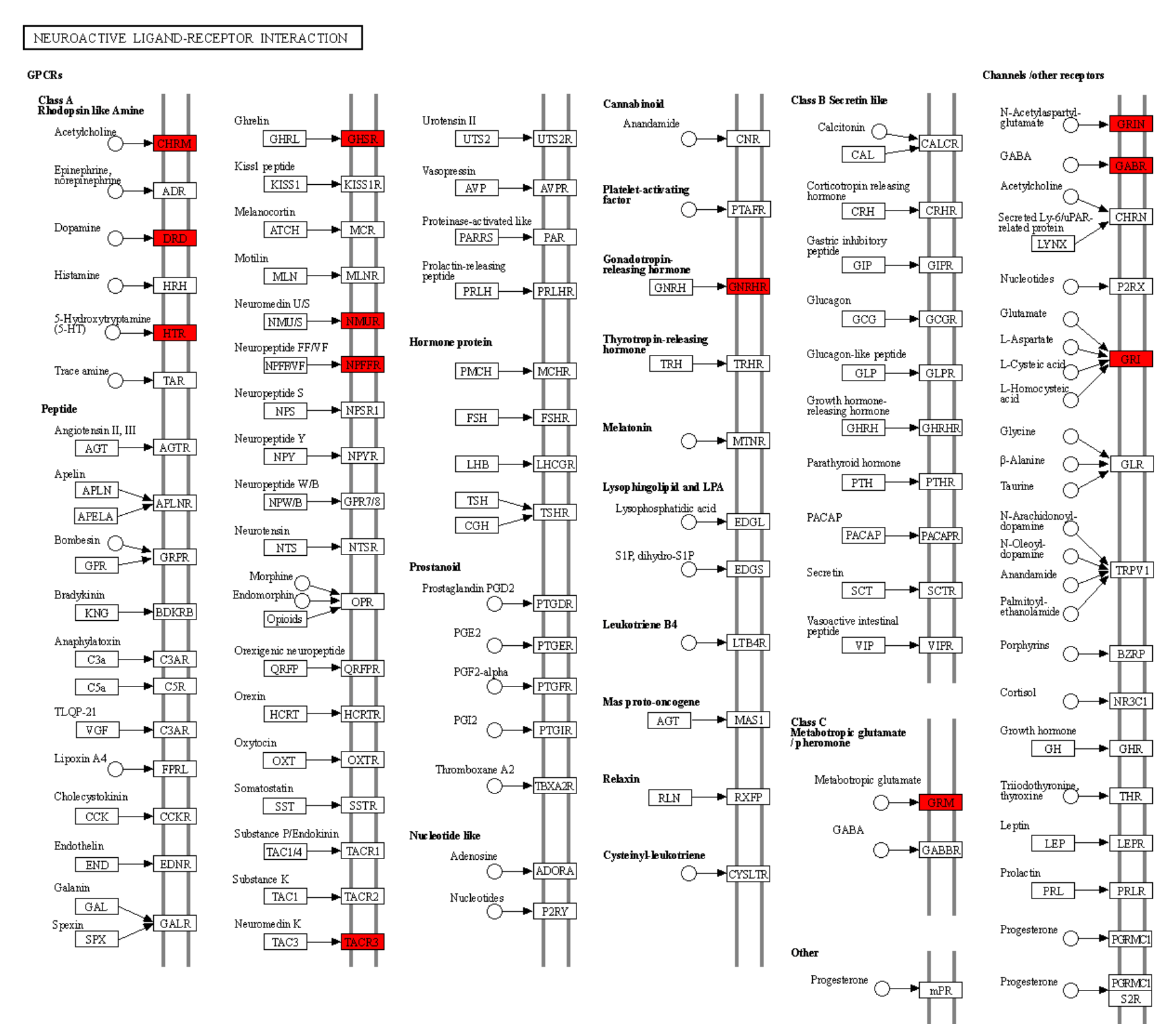

Supplement: Supplementary Figure 1 — GO enrichment analysis. Neuroactive ligand-receptor interaction category (KEGG). [file Image_1.tiff]
